# Selective Area Epitaxy of Quasi-1-Dimensional Topological Nanostructures and Networks

**DOI:** 10.3390/nano13020354

**Published:** 2023-01-15

**Authors:** Abdur Rehman Jalil, Peter Schüffelgen, Helen Valencia, Michael Schleenvoigt, Christoph Ringkamp, Gregor Mussler, Martina Luysberg, Joachim Mayer, Detlev Grützmacher

**Affiliations:** 1 Peter Grünberg Institute (PGI-9), Forschungszentrum Jülich, 52425 Jülich, Germany; 2 JARA-FIT (Fundamentals of Future Information Technology), Jülich-Aachen Research Alliance, Forschungszentrum Jülich and RWTH Aachen University, 52425 Jülich, Germany; 3 Peter Grünberg Institute (PGI-10), JARA-Green IT, Forschungszentrum Jülich, 52425 Jülich, Germany; 4 Ernst Ruska-Centre (ER-C) for Microscopy and Spectroscopy with Electrons, Forschungszentrum Juelich, 52425 Jülich, Germany; 5 Central Facility for Electron Microscopy (GFE), RWTH Aachen University, 52074 Aachen, Germany

**Keywords:** topological nanostructures, quasi-1D network, selective area growth, molecular beam epitaxy

## Abstract

Quasi-one-dimensional (1D) topological insulators hold the potential of forming the basis of novel devices in spintronics and quantum computing. While exposure to ambient conditions and conventional fabrication processes are an obstacle to their technological integration, ultra-high vacuum lithography techniques, such as selective area epitaxy (SAE), provide all the necessary ingredients for their refinement into scalable device architectures. In this work, high-quality SAE of quasi-1D topological insulators on templated Si substrates is demonstrated. After identifying the narrow temperature window for selectivity, the flexibility and scalability of this approach is revealed. Compared to planar growth of macroscopic thin films, selectively grown regions are observed to experience enhanced growth rates in the nanostructured templates. Based on these results, a growth model is deduced, which relates device geometry to effective growth rates. After validating the model experimentally for various three-dimensional topological insulators (3D TIs), the crystal quality of selectively grown nanostructures is optimized by tuning the effective growth rates to 5 nm/h. The high quality of selectively grown nanostructures is confirmed through detailed structural characterization via atomically resolved scanning transmission electron microscopy (STEM).

## 1. Introduction

Since their experimental discovery in 2007 [1,2,3,4], topological insulators (TIs) have been studied intensively via angle-resolve photoemission spectroscopy (ARPES) [5,6,7], scanning tunneling microscopy (STM) [8,9,10], transmission electron microscopy (TEM) [5,11], magneto-transport [12,13,14,15,16] and many more experimental techniques. Their Dirac surface states are topologically protected and have their spin orientation coupled to their momentum. This exotic property creates the basis for the use of TIs in future spintronics and quantum computing schemes [17,18]. After more than a decade of fundamental research, these materials are well understood and are ready to be implemented into the next generation of quantum devices.

Quasi-1D nanostructures of (Bi,Sb)_2_Te_3_ are the preferred device geometry, as the enhanced surface-to-bulk ratio at those scales favors highly spin-polarized transport via the topological surface states. Furthermore, quasi-1D nanostructures allow for the creation of localized Majorana zero modes [19,20,21,22] when proximitized by an s-wave superconductor, such as Nb or Al [23,24,25,26,27]. As promising as devices based on quasi-1D TIs may be, their proper fabrication is a challenge in itself. From a pure growth perspective, the most natural way of realizing topological quasi-1D structures of high quality is via vertical growth of nanowires by means of VLS [9,28,29,30], PVD/CVD [31,32,33,34,35,36] or molecular beam epitaxy (MBE) [37,38,39,40]. However, in order to exploit the physics of the surface states in topological devices, a holistic perspective on the fabrication process must be taken, e.g., from growth to nanofabrication to their transfer into a cryostat, where the devices may finally be operated.

While common nanostructures, such as exfoliated flakes and vertical nanowires, possess a high crystalline quality, they need to undergo conventional cleanroom fabrication to ensure they can be further refined into a topological device. This includes the exposure of ambient conditions and chemical solutions, which poses a massive threat to the devices, as their functionality is severely impaired by oxidation and degradation, as has been found in multiple studies [41,42,43,44,45,46]. 

In this work, a sophisticated study on selective area epitaxy (SAE) is presented that facilitates the realization of laterally grown topological nanostructures. The as-grown nanostructures are fully encapsulated under ultra-high vacuum conditions via a stencil mask that covers the side facets, and an *in situ* capping layer that is deposited on the top surface. This method allows the patterning of TI epilayers into arbitrary shapes having ultra-low dimensions, without any exposure to chemicals or air [15,16,47], which is almost impossible to realize via conventional lithography of planar TI films without degrading the crystal quality. The protective dielectric materials around the TI structure preserves its delicate surfaces at ambient conditions. Thus, this process not only prevents any structural or atomic defects originating from the etching processes, but also avoids the oxidation of TI’s surfaces at air. Optionally, with a precise alignment accuracy, the *in situ* fabrication of electrodes can also be implemented into this process [25]. Various devices, such as topological nanoribbons [47], nano-hallbars [16], multi-terminal junctions [15,48] and topological Josephson junction-based transmon qubit [49], etc., that include one or more ultra-high vacuum lithography steps, have already been fabricated and characterized. 

SAE is not a new technique. Previously, it has been realized for various material systems, such as III-V compound semiconductors [50,51,52,53], group IV semiconductors [54,55,56] and even carbon nanotubes [57]. SAE is a technique that facilitates epitaxial growth of the desired crystal locally on one of two surfaces belonging to different material systems. Therefore, it can only be achieved on a substrate exposing composite materials. For selectivity, one of these materials needs to favor the epitaxial growth, while the other material should prevent nucleations from stabilizing. The surface of the latter material is called the “blocking surface” and is often amorphous in nature. The surface targeted for the epitaxial growth is usually crystalline and is known as the “epitaxial surface”. SAE is a very temperature-sensitive technique, as it highly depends on the adsorption-to-desorption ratio (ADR), i.e., the sticking coefficient [58,59,60] of adatoms on the blocking vs. epitaxial surfaces. That is why it is impossible to adopt SAE for materials that either crystallize at low-temperatures, such as Bi and Sb, or exhibit a high sticking coefficient, such as Cr and Ti. However, it can easily be applied to materials that crystallize at relatively high temperatures, such as GaN [61,62]. 

The topological insulators investigated in this work are grown via MBE at moderate temperatures between 200 °C to 300 °C. This low temperature range signifies the importance of selecting the right blocking material for SAE of TIs. While reports on SAE of 3D TIs already exist, they mainly focused on the proof of principle without the comprehension of growth dynamics. Among them, one study discusses SAE of Bi_2_Te_3_ and Sb_2_Te_3_ on silicon-on-insulator (SOI) substrates. The proposed approach provides SAE at macroscale and is scalable; however, it does not facilitate the realization of nanostructures as the patterned mesa structures do not contain sidewalls to confine the TI epilayer in the desired shape and dimensions. The tendency of 3D TIs to grow laterally hinders the achievement of well-defined structures and sharp edges in the SOI platform. Eventually, the growth in nanostructures results in randomly shaped free-standing or suspended sheets of TI crystals, as identified by Lanius et al. [63].

In this study, a state-of-the-art SAE approach with an emphasis on understanding the growth dynamics at the nanoscale and on optimizing the crystal quality in selectively grown TI nanostructures, is demonstrated. Contrary to the SOI technique, this approach facilitates the precise fabrication of quasi-1D networks. It also implements additional requirements for integration of topological nanostructures into future device architectures, i.e., design-flexibility and scalability. Variations of straight nanostructures, such as constrictions or arbitrary branching, are possible and are demonstrated in this work. The scalability is validated by fabricating networks of selectively grown nanostructures with a high precision on substrates up to 4” wafers. These findings will provide the community all the necessary information to develop a platform that is capable of delivering fully *in situ* fabricated nanostructures using standard fabrication tools. Though, the approach presented in this work is developed focusing topological features, BiSbTe alloys are well known for their phase-change mechanism and thermoelectric characteristics. We hope that this platform will provide the respective communities significant fabrication capabilities to prepare fully *in situ* nanostructured devices for low-power energy applications. While the process is demonstrated on Si substrates, with minor modifications, it can also be adapted for other crystalline substrates, such as sapphire or SiC.

## 2. Fabrication of Patterned Substrates and Growth Optimization of Planar Films

**Figure 1:**
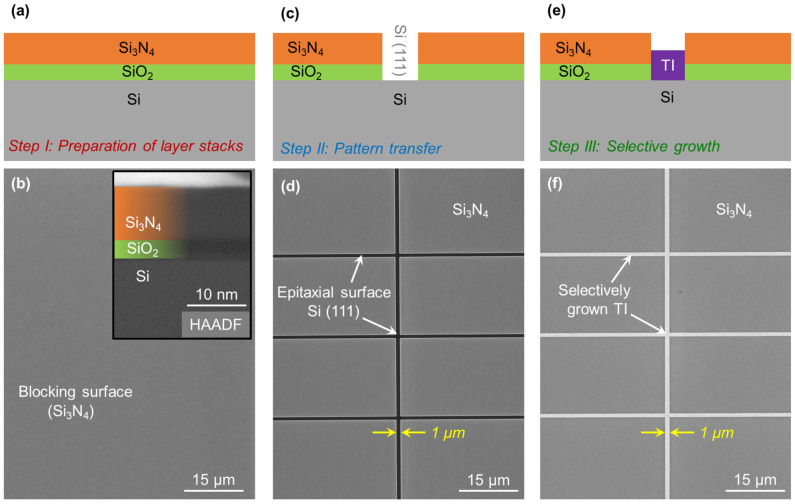
Illustration of major steps during SAE. The cross-sectional sketches including (**a**) the preparation of blocking surface on Si wafer (combinational layer stack of SiO_2_ and Si_3_N_4_), (**c**) pattern transfer and (**e**) the selective growth of TI on the exposed epitaxial surface. While (**b**,**d**,**f**) display the corresponding scanning electron micrographs (SEM) taken at the respective fabrication steps. The inset in (**b**) depicts the cross-section high angular-annular dark field (HAADF) image of the blocking layers acquired via scanning transmission electron microscopy (STEM).

In the first step, the pattern of the desired nanostructures is transferred onto the stencil mask. For this, the Si substrate must be covered with the blocking material of choice. Various amorphous materials including Al_2_O_3_, HfO_2_, SiO_2_ and Si_3_N_4_ are tested. Taking the thermal treatment during epitaxy and reproducibility of the fabrication steps into account, SiO_2_ and Si_3_N_4_ are found to be the most suitable candidates. Both, however, come with their own unique challenges. Due to isotropic etching in HF based solutions, SiO_2_ does not provide the desired dimensional integrity at the nanoscale. The resulting pattern has been observed to widen, and the blocking layer diminishes in thickness, forming pinholes. Si_3_N_4_, on the other hand, exerts tensile strain onto the Si interface [64,65,66,67] that may introduce unintended defects in the resulting epilayer. Individually, neither SiO_2_ nor Si_3_N_4_ can be utilized as a reliable blocking material; however, a combinational stack containing a thin layer of SiO_2_ at the interface with Si (111) followed by a relatively thick Si_3_N_4_ layer provides a solution for a functional blocking surface. Thereby, SiO_2_ acts as a buffer and counters the strain between Si (111) and Si_3_N_4_, while the stability of Si_3_N_4_ in HF solution provides the dimensional integrity at the nanoscale. After the successful coverage of the epitaxial surface with the combined layer stack, the desired patterns are transferred to the substrate using e-beam lithography and CHF_3_-based reactive-ion etching of Si_3_N_4_. Thus, trenches in Si_3_N_4_ are formed to expose the buried Si (111) surfaces that are still covered with a thin layer of SiO_2_. Before epitaxy, the substrate is treated with HF solution to release the buried epitaxial surfaces of Si (111) and to passivate the Si dangling bonds with hydrogen [68]. Later, SAE is performed in the transferred patterns. Figure 1 illustrates three major steps involved in realizing SAE from the preparation of the blocking surfaces to the selectively grown crystal. In addition, several examples of patterned substrates can be seen in Figure S2.

After the successful fabrication of patterned substrates and before SAE, the optimum growth conditions for planar TI films are evaluated on plain Si substrates. A systematic study focusing, at first, on the binary TIs i.e., Bi_2_Te_3_ and Sb_2_Te_3_ and, later, extended to the ternary alloy Bi_x_Sb_2−x_Te_3_, commonly referred to as BST, is conducted. Details on the growth optimization can be found in supplementary Figures S3–S5. Various parameters, such as the surface treatment and surface passivation, etc., are found to impact the crystal quality of the final epilayer. The two key parameters, however, are the substrate/growth temperature (*T_sub_*) and the growth rate of planar thin films (*R_TF_*). 

**Figure 2:**
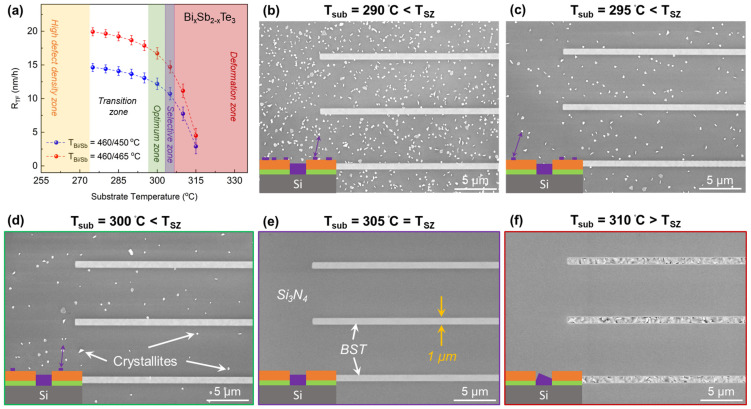
The effects of growth temperature (*T_sub_*) on planar and selectively grown TI films. (**a**) Temperature-dependent growth of BST planar films, where the sets of red and blue data points represent growths conducted at relatively high and low *R_TF_*, respectively. Based on XRD, the entire *T_sub_* range is divided into high defect density (yellow), transition (white), optimum (green) and deformation (red) temperature zones. The optimum zone is found to provide the highest crystal quality. During SAE on templated substrates, a finite temperature range is identified that provides perfect selectivity, along with a high crystal quality. This *T_sub_* range is named “selective temperature zone (T_SZ_)” and is highlighted with the purple color. SAE of BST crystal conducted outside the selective zone at (**b**) *T_sub_* = 290 °C, (**c**) *T_sub_* = 295 °C and (**d**) *T_sub_* = 300 °C, indicating non-perfect selectivity due to presence of crystallites on the blocking surface (*T_sub_* < T_SZ_). (**e**) Growth conducted at *T_sub_* = 305 °C exhibits perfect selectivity, (**f**) SAE conducted at *T_sub_* = 310 °C also exhibits perfect selectivity; however, the crystal starts to deform due to low ADR (*T_sub_* > T_SZ_).

Epilayers were prepared over *T_sub_*, ranging from 250 to 320 °C. The structural characterization of the as-grown epilayers was performed via X-ray diffraction (XRD). The acquired full width at half maximum (FWHM) value from the rocking curve (*Δω*) analysis of the (0 0 15) peak is utilized as the qualitative figure of merit to compare epilayers prepared with different growth parameters. Based on *Δω* analysis, the entire *T_sub_* range is categorized into distinct zones, including the high defect density (yellow), the transition (white), the optimum (green) and the deformation zone (red) in Figure 2a. As the names indicate, the crystal quality is observed to be poor (FWHM > 500″) in the high defect density zone. The crystal quality continuously improves with increasing *T_sub_* throughout the transition zone. The optimum zone corresponds to a set of *T_sub_* that resulted in epilayers with FWHM ≤ 150″. The optimum zone is found to be quite narrow, with *T_sub_* = 300 ± 5 °C. The deformation zone indicates *T_sub_* values at which the desorption rate of adatoms supersedes the adsorption rate (ADR < 1). The initial stages of this growth regime result in a non-coalesced epilayer. Further increase in *T_sub_* results in no growth of epilayer as all incoming adatoms desorb from the epitaxial surface (ADR = 0).

After identifying an optimal temperature window for thin film epitaxy, the crystal quality dependency on *R_TF_* is investigated. While keeping *T_sub_* fixed at the optimum value of 300 °C, *R_TF_* is varied between 2 nm/h to 20 nm/h. Lower values of *R_TF_* were found to reduce the density of extended defects (grains, domains, etc.) in the resulting epilayer providing a FWHM as low as 60″. Additionally, epilayers grown with *R_TF_* ≤ 5 nm/h show entirely suppressed rotational twin domains as confirmed via XRD *φ*–scan of the crystal (1 0 5) peak, the results of which can be seen in Figure S5. After finding optimal *T_sub_* and *R_TF_* for planar thin films, the findings are adapted to enable the selective growth of 3D TIs on patterned macrostructures. 

## 3. Selective Area Epitaxy

As the best crystal quality of the planar TI films is obtained in the optimum zone, the aim is to acquire selective growth within the same temperature window. At *T_sub_* = 300 °C, the beam fluxes of Bi, Sb and Te are adjusted to target *R_TF_* = 5 nm/h and are directed to the pre-patterned substrate. The growth of BST epilayers is successfully achieved in the etched trenches where the Si (111) epitaxial surfaces are exposed. The blocking surface, on the other hand, exhibited a partial selectivity with the formation of randomly distributed crystallites on Si_3_N_4_ surface, as depicted in Figure 2d. This indicates that the ADR of adatoms on the blocking surface is still finite, and *T_sub_* must be increased to acquire the perfect selectivity. This observation is confirmed via further degraded selectivity (high density of crystallites on the blocking surface) at reduced *T_sub_* of 290 °C and 295 °C, as depicted in Figure 2b,c. Therefore, *T_sub_* is increased in gradual steps of 1 °C and, eventually, at *T_sub_* = 305 °C, a perfect selectivity is achieved where the growth of BST crystals is attained only on the epitaxial surfaces inside the etched trenches, depicted in Figure 2e. The SAE with increasing *T_sub_* is further investigated until the deformation zone is reached as shown in Figure 2f. Thus, the temperature range that facilitates the perfect selectivity is identified and named “the selective zone (T_SZ_)”. T_SZ_ lies within the optimum zone of planar growth and marked with a purple-colored region in Figure 2a. As T_SZ_ is very narrow, it is highly important to precisely tune *T_sub_* to acquire the perfect selectivity. It has also been identified that the optimum and selective temperature zones differ for each TI material. Examples of Bi_2_Te_3_ and Sb_2_Te_3_ as shown in Figure S7. 

### Challenges at Nanoscale

**Figure 3:**
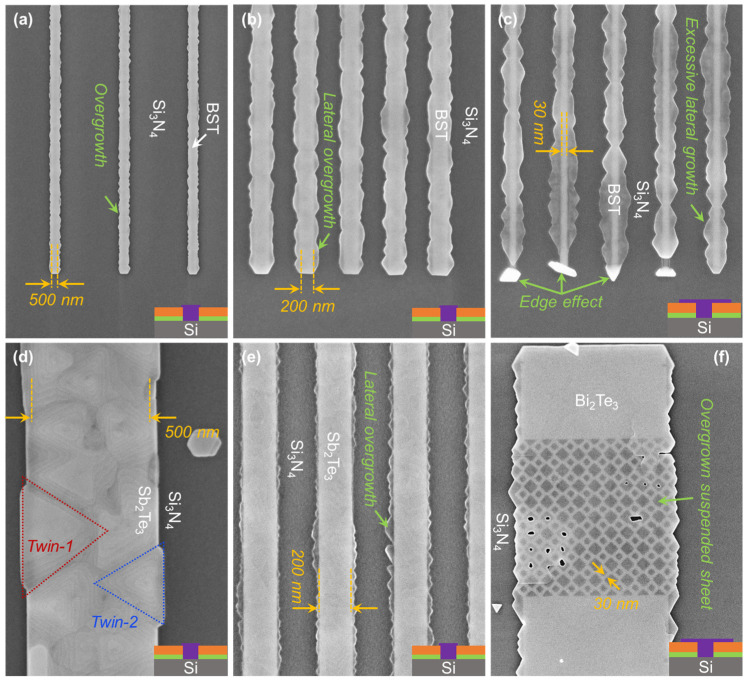
Pattern dimensions vs. effective growth rate (*R_eff_*). SEM images of SAE of BST alloy in nano-trenches with (**a**) *W* = 500 nm and (**b**) *W* = 200 nm exhibits overgrowth, with lateral extension of the layer on the blocking surface, and (**c**) *W* = 30 nm exhibits excessive lateral overgrowth and deformations at the edges confirming *R_eff_* >> *R_TF_*. The effects of enhanced *R_eff_* are also witnessed during SAE of Sb_2_Te_3_, where trenches with (**d**) *W* = 500 nm demonstrate high surface roughness and the formation of rotational domains. (**e**) For a reduced width of *W* = 200 nm, lateral overgrowth is observed. The effects of elevated *R_eff_*, when dimensions of both *W* and *L* go towards the nanoscale regime, can be observed in (**f**) where Bi_2_Te_3_ overgrowth results in the formation of a suspended sheet of TI layer.

So far, the patterned substrates contained only macrostructures (dimensions ≥ 1 μm), as depicted in Figure 2b–f. The acquired results have shown no apparent difference in morphology or thickness of the selectively grown layer, with respect to the planar film discussed earlier. As dimensions are scaled down to the quasi-1D limit, apparent changes in nanostructures to macrostructures are observed. While perfect selectivity is also achieved in the nanostructures, differences in the effective growth rate (*R_eff_*), dependent upon the pattern dimensions, are witnessed. Considering the trench depth and the thin film equivalent growth rate, also applied for SAE of macrostructures in Figure 2, the growth duration is adjusted to acquire the nano-trenches half-filled. The trenches, with dimensions in the nanometer range, targeted to be half-filled, are unexpectedly observed to be entirely filled as depicted in Figure 3. This clearly indicates that the effective growth rate (*R_eff_*) inside the nano-trenches is higher than the applied rate (*R_eff_* >> *R_TF_*). Moreover, this effects is observed to vary with dimensional changes of the trench pattern. For example in 500 nm-wide structures, the trenches are observed to be entirely filled, as shown in Figure 3a, whilst, for a width of 200 nm, the epilayers are observed to start growing laterally after filling the trenches, exceeding the boundary of the sidewalls, as demonstrated in Figure 3b. Reducing the dimensionality further to 30 nm wide trenches, excessive lateral growth of the epilayer is witnessed, as shown in Figure 3c. This phenomenon, i.e., *R_eff_* >> *R_TF_*, is not only observed during SAE of BST, it has also been witnessed for Sb_2_Te_3_, as seen in Figure 3d,e and Bi_2_Te_3_, shown in Figure 3f. The enhanced *R_eff_* during SAE of Sb_2_Te_3_ in a 500 nm-wide trench did not only introduce high surface roughness, but also degraded the crystal quality with a clear presence of twin defects. Analogous to planar epilayers prepared with *R_TF_* > 5 nm/h, SAE with a high *R_eff_* results in the formation of rotational twin domains. SAE of Bi_2_Te_3_ in ultra-low dimensional structures led to the formation of excessive lateral growth that resulted in the formation of a suspended TI sheet supported by TI pillars grown inside the trenches, as depicted in Figure 3f. Some additional defects in ultra-low dimensions, such as the edge effect (see Section 4), are also observed and indicated in Figure 3c. All these defects must be addressed before a reliable growth of nanostructures can be realized.

## 4. Growth Model 

**Figure 4:**
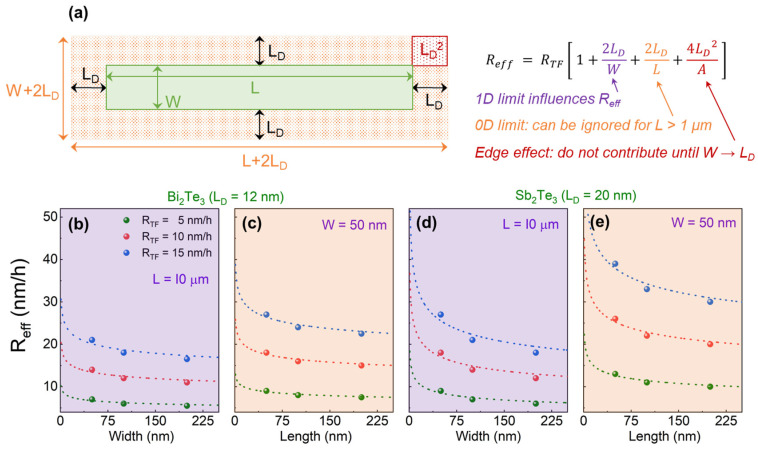
Selective growth model for calculating *R_eff_*. (**a**) A model representation explaining the impact of lateral diffusion length of adatoms (*L_D_*) on *R_eff_*, where a trench with a given surface area (A=W×L) experiences the effective influx of adatoms from an area of $\;A_{eff}=\left(W+2L_{D}\right)\left(L+2L_{D}\right)$. Evaluation of *L_D_* for Bi and Sb adatoms from the measured and fitted values of *R_eff_* for different applied *R_TF_*, i.e., 5 nm/h, 10 nm/h and 20 nm/h represented by green, red and blue data points, respectively, are shown in (**b**–**e**). The effects of trench width on resulting *R_eff_* values for both Bi_2_Te_3_ and Sb_2_Te_3_ can be seen in (**b**,**d**). The measured values of *R_eff_* are represented by the color dots, while the dotted lines in the corresponding colors represent fitting according to the Equation (2) with *L_D_* = 11.9 nm and 19.8 nm for Bi and Sb, respectively. The drastic enhancement in *R_eff_* with continually decreasing *L* of the structure while *W* is kept constant at 50 nm for (**c**) Bi_2_Te_3_ and (**e**) Sb_2_Te_3_. Here, the fitting is achieved according to Equation (3).

It is evident that *R_eff_* >> *R_TF_* for SAE at the nanoscale. Therefore, to realize high-quality SAE of nanostructures, *R_TF_* needs to be adjusted in such a way that *R_eff_* ≤ 5 nm/h. This parameter ensures the least defect density of the grown epilayer, as shown in Figures S4 and S5. To gain control over *R_eff_*, a statistical study with hundreds of selective growths of binary TIs, with pattern dimensions ranging from 100 μm down to 30 nm, is conducted. Based on this study, a growth model is developed to formulize how *R_eff_* depends on both material and dimensional parameters. SEM observations have confirmed that *R_eff_* scales inversely proportional to the surface area of the nanostructure, however for a given area, the perimeter also influences *R_eff_*. The lateral diffusion length of the adatoms (*L_D_*) on the blocking surface has been found to be the most important factor that impacts *R_eff_*. The adatoms close to the pattern boundaries diffuse laterally on the blocking surface and contribute to enhance the growth rate of the TI epilayer inside the trench. As a consequence, the accumulative flux within this effective area (*A_eff_*) on the blocking surface in close proximity to the trench contributes to the growth of the TI inside the trench. Hence, within the actual area (A), defined by the geometry of the trench, enhanced effective growth rates (*R_eff_* > *R_TF_*) are observed. For simplicity, refer to Figure 4a where the actual area of the trench is depicted with green color, whilst the effective area (*A_eff_*) is highlighted with orange dotted region, enclosing the actual area (*A*). This relation can be summarized in form of Equations (1a–c), where *W* and *L* are the structure’s width and length.

(1a) $$\frac{R_{eff}}{R_{TF}}=\frac{A_{eff}}{A},$$

(1b) $$R_{eff}=R_{TF}\left[\frac{\left(L+2L_{D}\right)\left(W+2L_{D}\right)}{W\times L}\right],$$

(1c) $$R_{eff}=R_{TF}\left(1+\frac{2L_{D}}{W}+\frac{2L_{D}}{L}+\frac{4L_{D}^{2}}{W\times L}\right).$$

In this study, *L* is kept in the micrometer range ensuring $2L_{D}/L<<1$ and, therefore, negligible. Moreover, the term $\;4L_{D}^{2}/A$, in most cases, is so small that it does not have considerable impact on *R_eff_*. The approximate formula for quasi-1D trenches can be simplified to:

(2) $$R_{eff}=R_{TF}\;\left(1+\frac{2L_{D}}{W}\right).$$

Hence, for quasi-1D trenches, the vital parameters affecting *R_eff_*, other than *R_TF_*, are *W* and *L_D_*. To calculate *R_eff_* for a certain composition, we first need to evaluate L_D_ of elements which determine the growth rate, i.e., Bi and Sb as Te flux has been found to have no effect on the growth rate, discussed in Figure S6. *L_D_* of rate-controlling elements, i.e., Bi and Sb, is extracted from the measured value of *R_eff_*, using Equation (2). *R_eff_* is determined from the growth duration and the measured thickness of the epilayer in the trench using AFM line scans before and after SAE, as shown in Figures S9–S12. In some cases, the thickness of the epilayer is also confirmed through analyzing the epilayer cross-section using STEM after the samples were prepared via focused ion-beam (FIB). An example is shown in Figure S8. Thus, *L_D-Bi_* and *L_D-Sb_* are extracted using the growth of binary TIs. *L_D-Te_*, however, cannot be measured as the Te flux is always supplied in excess during the epitaxy of 3D TIs. It can only be measured when the Te flux will start to impact *R_TF_*. *L_D-Te_* has been extracted from SAE of Te-based stoichiometric alloys, such as Bi_1_Te_1_, Bi_4_Te_3_ and Sb_1_Te_1_, etc., which is outside the scope of this work [69]. 

The extracted values have indicated that Bi has much smaller lateral diffusion length (*L_D-Bi_* = 12 ± 0.5 nm) than Sb (*L_D-Sb_* = 20 ± 0.5 nm) on the Si_3_N_4_ surface, which differs from what is observed on Si (111), as reported in an earlier study [11]. In ternary BST alloys, the value of the averaged *L_D_* (this is the value which enters Equation (2)) depends upon the exact stoichiometry of the material. For example, in the case of Bi_1_Sb_1_Te_3_, the effective values of *L_D_* will be the average of Sb and Bi with *L_D_* = 16 nm > *L_D-Bi_*, which will increase to 19.6 nm for Bi_0.1_Sb_1.9_Te_3_. This is why BST nanostructures show higher *R_eff_* compared to SAE of Bi_2_Te_3_. Please note, that here only the enhancement of *R_eff_* is considered. Due to the difference in *L_D_* of Bi and Sb, the stoichiometry within the trenches will slightly change and nanostructures will exhibit higher Sb contents compared to the thin films that are grown with identical fluxes. 

Equation (2) holds true if *L* > 1 μm; however, as soon as the length of the pattern drops below 1 μm, the assumption $2L_{D}/L<<1$ is no longer valid. In that case, *R_eff_* can be described using Equation (3), and the resulting changes in *R_eff_* at nanoscale occur more rapidly. An example of such a change in *R_eff_* can be seen in Figure 3f where, despite the relatively low *L_D-Bi_*, an excessive overgrowth is witnessed as both dimensions, *W* and L, reach the nanoscale.

(3) $$R_{eff}=R_{TF}\left[1+2L_{D}\left(\frac{1}{W}+\frac{1}{L}\right)\right].$$

The *L_D_* values for Bi and Sb are re-evaluated and confirmed by taking all dimensions into account. Figure 4b depicts the trend in *R_eff_* during SAE of Bi_2_Te_3_, when the trench width (*W*) is reduced from 250 nm down to 50 nm. The effect of varying *R_TF_* can also be observed. The circular points represent the measured *R_eff_* values from the selectively grown nanostructures, while the dotted lines in the corresponding colors represent the fitting of these measured values according to the model presented in Equation (2), where the length scale (1/L<<1) is ignored. With this information, the diffusion length is confirmed to be *L_D-Bi_* = 11.9 ± 0.2 nm. Similarly, Figure 4d represents the information for Sb_2_Te_3_ confirming *L_D-Sb_* = 19.8 ± 0.2 nm. The trend in *R_eff_* with the decreasing pattern length (*L*) is also measured and depicted in Figure 4c,e for Bi_2_Te_3_ and Sb_2_Te_3_, respectively. In order to observe the effect more prominently, the trench width is fixed at 50 nm, while the trench length is gradually decreased from 250 nm to 50 nm. The dotted lines represent the fitting of the measured values according to Equation (3). It can be seen that the decline in both structural parameters (*W* and *L*) simultaneously impacts *R_eff_* more vigorously and pushes it as high as $\;R_{eff}\geq 3\times R_{TF}$, as shown in Figure 4e. The values of *L_D_* obtained from the fitting of the curves to our predicted model agree well with the *L_D_* calculated from the measured thickness of the epilayer inside the trenches. Thus, the model provides an accurate estimate for any set of pattern dimensions during SAE of Bi, Sb and Te-based TIs. The model is further developed for nonlinear and circular patterns, and the resulting analytical expressions are formulated, shown in Figures S13 and S14. Before proceeding to the optimization of nanostructures, the edge effect, depicted in Figure 3c, which has not been taken into account yet, is discussed. During the evaluation of *R_eff_*, the term $4L_{D}^{2}/A$ in the Equation (1c) was ignored as it acts marginally on *R_eff_*. It holds true as long as the smallest dimensional parameter remains larger than $\;2L_{D}$. Once this limit is crossed, the term $4L_{D}^{2}/A$ cannot be ignored anymore. It influences *R_eff_* at the corners, resulting in the formation of crystallites and other defects due to excessively high *R_eff_* reaching $\geq 5\times R_{TF},$ as witnessed in Figure 3c with *W* = 30 nm and *L_D_* = 16 nm (BST).

## 5. Optimization of Nanostructures 

The analytical model, as mentioned earlier, provides an accurate estimation of *R_eff_* that allows for the adjustment of growth parameters to given geometries of nanostructures, avoiding vertical overgrowth and lateral extension of the layer above the sidewalls. However, despite obtaining selectively grown structures, the crystal may still exhibit high defect density as *R_eff_* >> 5 nm/h. The growth model can also be used to determine an *R_TF_* that facilitates SAE of defect-free nanostructures with *R_eff_* ≤ 5 nm/h. An example of such an evaluation for Sb_2_Te_3_ crystal (*L_D_* = 20 nm) is described in Equation (4) for a pattern having a width and length of 50 nm and 10 μm, respectively.

(4) $$5\;\frac{nm}{h}=R_{TF}\;\left(1+\frac{2\times 20}{50}+\frac{2\times 20}{1000}\right)\;\Rightarrow \;R_{TF}=2.7\;\frac{nm}{h}.$$

**Figure 5:**
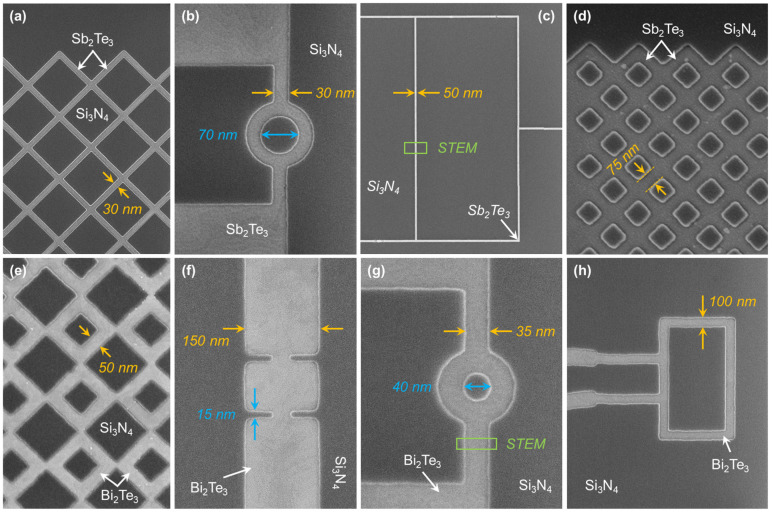
Examples of SAE with controlled *R_eff_* = 5 nm/h, conducted on different substrates where *R_TF_* is adjusted according to the dimensional parameters. Scanning electron micrographs (SEM) of selectively grown nanostructures of Sb_2_Te_3_ (**a**–**d**) and Bi_2_Te_3_ (**e**–**h**) reaching quasi-1D limits confirming the possibility of achieving a scalable network of topological nanostructure adoptable to arbitrary layouts.

Hence, for a pattern with dimensions *W* = 50 nm and *L* = 1 μm, the applied growth rate (*R_TF_*) must not exceed 2.7 nm/h. Utilizing our model, the defect-free selective growth at *R_eff_* = 5 nm/h for various structures ranging in dimensions from μm to nm scale, is performed where the required values of *R_TF_* are adjusted to the dimensions of the layout of the chip. By adjusting *R_eff_* = 5 nm/h, according to the layout of the chip containing quasi-0D structures, even the edge effect can be fully suppressed. SEM images of several examples of the controlled SAE in nanostructures are shown in Figure 5a–d for Sb_2_Te_3_ and Figure 5e–h for Bi_2_Te_3_, where the homogeneous thicknesses and smooth surfaces of the selectively grown nanostructures are evident. 

### 5.1. Multi-Dimensional Structures 

Utilizing the analytical model, the modifications in *R_TF_* to acquire *R_eff_* = 5 nm/h is not complex. However, if a pre-patterned substrate contains structures with a variety of dimensions ranging from μm to nm scale, the selection of a suitable *R_TF_* value can be challenging as *R_eff_* varies with the changing dimensions of the pattern. For instance, *R_eff_* can scale by a factor of 3 of the applied *R_TF_* in very narrow trenches, as observed in Figure 3f, while it remains effectively unchanged at the micrometer scale (*R_eff_* ≈ *R_TF_*). For example, if *R_TF_* is optimized to achieve *R_eff_* = 5 nm/h for a structures having *W* = 1 μm, the defect-free growth should be achieved in all macrostructures; however, the nanostructures will experience the elevated *R_eff_* and will exhibit crystal defects and may also result in overgrown trenches, just as observed in Figure 3. On the other hand, if *R_TF_* is tuned to achieve *R_eff_* = 5 nm/h for a structure having *W* = 30 nm, the high crystal quality nanostructures will be achieved while macrostructures will exhibit the non-coalesced epilayers with crystal defects, due to significantly reduced growth rates and low ADR. This limitation applies only if the pre-patterned substrate contains structures with a vast range of dimensions. A solution to this problem is to segregate the macro- and nanostructures on different substrates and to conduct SAE separately for each chip with the corresponding modified *R_TF_* to avoid any crystal defects. 

**Figure 6:**
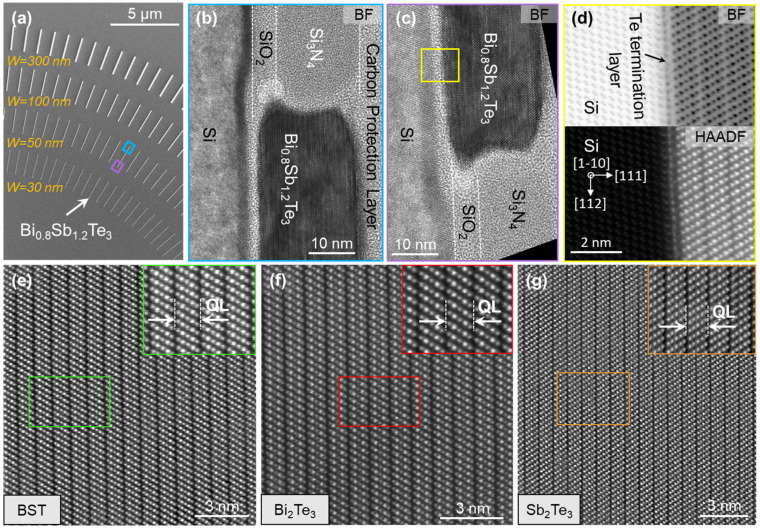
Dimensional dependence of *R_eff_* during SAE. (**a**) An example of BST alloy where *R_TF_* is adjusted to achieve *R_eff_* = 5 nm/h for trenches with *W* = 50 nm. The patterned substrate contains structures of varying sizes (*W* = 30 nm, 50 nm, 100 nm and 300 nm), which results in different thicknesses for each structure. (**b**,**c**) Structural characterization of the BST layer cross-section via TEM where lamellae are extracted at both ends of the nanoribbon marked in (**a**). (**d**) Atomic resolution HAADF and bright field (BF) STEM images acquired at the interface between Si substrate and BST epilayer, where the presence of a Te monolayer terminating the Si dangling bonds can be observed. The HAADF images in the bulk region of (**e**) BST, (**f**) Bi_2_Te_3_ and (**g**) Sb_2_Te_3_ are acquired.

An example of this interesting phenomenon, i.e., the dimensional dependence of *R_eff_*, can be observed in Figure 6a, where the pre-patterned substrate contains structures of varying sizes. The *R_TF_* is optimized to achieve *R_eff_* = 5 nm/h of BST in 50 nm-wide trenches; however, 30 nm and 300 nm wide structures are also included and have the corresponding values of 6.25 nm/h and 3.44 nm/h, respectively. This variation in *R_eff_* has led to a difference in the obtained thickness of the nanostructures from 20 nm (*W* = 30 nm) to almost 11 nm (*W* = 300 nm) after the growth duration of 3 h. This example highlights the extent of deviation in *R_eff_*, and resulting thicknesses of the epilayers during SAE, if the patterned substrate contains structures of varying sizes. However, as *R_TF_* was modified according to 50 nm-wide trench, all dimensions have exhibited fully-coalesced and smooth growth of nanostructures, which would not have been possible if the substrate would have contained macrostructures.

### 5.2. Structural Characterization

Due to the small dimensions, structural investigation of selectively grown nanostructures is not possible via XRD. Therefore, structural characterizations are performed via TEM/STEM. The technical details of the system used for these investigations can be found in supplementary Section S6. For TEM characterization, lamellae of 50 nm wide nanoribbon, marked in Figure 6a, are extracted along Si [1−10] orientation. Figure 6b,c depict the bright field TEM images of both ends of the nanoribbon exhibiting the fully coalesced and smooth layers of BST. With the non-aberration corrected TEM, the investigation of high resolution atomic-interface is challenging which increases in complexity if the elements exhibit a high difference of Z-values (atomic number) such as Si and TI elements.

An advantage of aberration corrected HAADF image acquired via STEM is its correlation with the neighboring atoms where a high difference of Z-values offers the advantage to differentiate between elements. Figure 6d depicts both the bright field (BF) and HAADF images of the interface between Si substrate and BST quintuple layers where a Te monolayer terminates the Si dangling bonds and, hence, provides a platform for the van der Waals epitaxy. Moving away from the interface, the central regions of BST, Sb_2_Te_3_ and Bi_2_Te_3_ are investigated and depicted in Figure 6e–g. The corresponding lamellae of Sb_2_Te_3_ and Bi_2_Te_3_ are extracted from the marked regions in Figure 5. HAADF images confirm the high crystal quality of the selectively grown nanostructures without the presence of any structural defects (domains, stacking faults and dislocations), as the epilayers are prepared with *R_eff_* = 5 nm/h.

## 6. Conclusions

In this work, it is shown how to optimize the crystal quality of selectively grown nanostructures and networks of (Bi,Sb)_2_Te_3_ topological insulators. The presented technique meets the demands for exploiting quasi-1D topological nanostructures in advanced quantum devices. The demonstrated fabrication processes are easy to implement and only require conventional CMOS technology. They prevent critical material issues, such as surface oxidation and structural degradation due to chemical exposure, via fully *in vacuo* patterned and encapsulated structures of ultra-low dimensions. The ability to realize complex and scalable design architectures is the key advantage of the presented technique.

The development of an analytical growth model enables control over selective growth by linking pattern dimensions to the growth parameters. Applying this model allows to transfer the knowledge of high-quality macroscopic planar films to the growth of defect-free nanostructures. However, our model suffers from one limitation, i.e., achieving high crystal quality for structures of different dimensions in one growth run. This limitation, however, can be bypassed by segregating the nano and mesoscopic patterns on different substrates, if possible.

We hope that our findings will stimulate further research on fully *in situ* fabricated devices on Si and other substrates, e.g., sapphire or SiC. For the future, it is planned to study the difference in diffusion lengths of elements on the blocking surface and the dimensional-dependent effective growth rates in selectively grown ternary and quaternary alloys on various substrates. These experiments can provide additional insights and help to further refine our model.

## Data Availability

The data presented in this study are openly available in RWTHData at [http://dx.doi.org/10.18154/RWTH-2022-06227].

## Supplementary Materials

The following supporting information can be downloaded at: https://www.mdpi.com/article/10.3390/nano13020354/s1, Figure S1. RTA timeline representing all three process steps including ramp-up, bake-out and cool-down of Si_3_N_4_ annealing process. Figure S2. SEM images of several patterned substrates where the dark contrast represents the epitaxial surfaces inside the etched trenches after removing Si_3_N_4_ via RIE and SiO_2_ via HF solution while the bright contrast represents the blocking surfaces. Figure S3. Growth rate (*R_TF_*) vs. *T_sub_* behavior of (a) Bi_2_Te_3_ and (b) the corresponding FWHM values of the rocking curve acquired at the (0 0 15) peak of the crystal. The Yellow, white, green and red colors represent the high defect density, transition, optimum and deformation zones respectively. Figure S4. XRD *2θ-ω* scans of approx. 30 nm thick epilayers of (a) Bi_2_Te_3_, (b) Sb_2_Te_3_ and (c) Bi_0.5_Sb_1.5_Te_3_ alloy. The relative intensity of the (0 0 9) diffraction peak in BST alloy (c) indicates the presence of higher Sb contents. (d) Displays the rocking curve (*Δω*) scan of Bi_2_Te_3_, acquired at the (0 0 15) peak, along with Gaussian fitting. Figure S5. Analysis of rotational twin domains via XRD φ-scan for Bi_2_Te_3_ epilayers acquired at the (1 0 5) peak along with their relative intensity collinear with Si (311) and (220) orientations. (a) Epilayers prepared at higher *R_TF_* exhibited high density of twin defects. (b) Suppressed twin domains along Si (220) with the relative abundance of 1:9 is observed in epilayers that were prepared at moderate *R_TF_*. (c) Twin free epilayers collinear only with Si (311) are obtained when prepared at low *R_TF_*. Figure S6. The trend of average surface roughness with changing Te flux. The red zone indicates the critical *T_Te_* where Bi/Te or Sb/Te flux ratios cross the limit and result in the altered stoichiometry. The green zone indicates the lowest possible *T_Te_* that provides the best (lowest) surface roughness while keeping the stoichiometry intact. Figure S7. Growth rate (*R_TF_*) vs. *T_sub_* behavior of (a) Bi_2_Te_3_, (b) Sb_2_Te_3_ for the identification of optimum and selective temperature zones. Figure S8. STEM-HAADF images of (a) Bi_2_Te_3_ and (b) Sb_2_Te_3_, acquired at the cross-section of 500 nm wide selectively grown nanoribbons along Si [1−10] orientation. Figure S9. AFM topographical images of selectively grown crystals of (a) Sb_2_Te_3_ and (b) Bi_2_Te_3_ on the Si (111) pre-patterned substrates. Figure S10. The topographic line scans measured via AFM tapping mode in a 500 nm wide structure before and after the SAE of Bi_2_Te_3_ that provide the quite accurate measurement of the layer thickness. Figure S11. The topographic line scans measured via AFM tapping mode in a 100 nm wide structure before and after the SAE of Sb_2_Te_3_. Figure S12. The topographic line scan measured via AFM tapping mode in a 30 nm wide structure after the SAE of the BST alloy that indicates the excessive overgrowth in the nanostructure. Figure S13. Schematics of a circular trench and the representation of area before and during the selective growth (*A_eff_*) due to lateral diffusion length of adatoms (*L_D_*). The radius *R* of the object increased to (*R+L_D_*) while evaluating *A_eff_* during SAE. Figure S14. Schematics of a circular ring trench and the representation of area before and during the selective growth (*A_eff_*) due to lateral diffusion length of adatoms. The radii of external *R_1_* and internal rings *R_2_* of the ring structure will increase to *R_1_+L_D_* and *R_2_-L_D_* respectively while evaluating *A_eff_* during SAE. References [16,69,70,71] are cited in Supplementary Materials.
