# Supplementary material for: Selective Area Epitaxy of Quasi-1-Dimensional Topological Nanostructures and Networks
Source: Nanomaterials (Basel). 2023 Jan 15;13(2):354. doi: 10.3390/nano13020354 (PMC9863233; doi:10.3390/nano13020354)
Supplement: Supplementary file 1 [file nanomaterials-13-00354-s001.zip › nanomaterials-2164300-SI.pdf]

nanomaterials

MDPI

Supplementary Material

Selective Area Epitaxy of Quasi-1-Dimensional Topological Nanostructures and Networks

Abdur Rehman Jalil 1,2,3,* , Peter Schüffelgen 1,2, Helen Valencia 2,4, Michael Schleenvoigt 1,2 , Christoph Ringkamp 1,2, Gregor Mussler 1,2, Martina Luysberg 4, Joachim Mayer 4,5 and Detlev Grützmacher 1,2,3

1 **Peter Grünberg Institute (PGI-9), Forschungszentrum Jülich, 52425 Jülich, Germany**

2 **JARA-FIT (Fundamentals of Future Information Technology), Jülich-Aachen Research Alliance,**

Forschungszentrum Jülich and RWTH Aachen University, 52425 Jülich, Germany

3 **Peter Grünberg Institute (PGI-10), JARA-Green IT, Forschungszentrum Jülich, 52425 Jülich, Germany**

4 **Ernst Ruska-Centre (ER-C) for Microscopy and Spectroscopy with Electrons, Forschungszentrum Juelich,**

52425 Jülich, Germany

5 **Central Facility for Electron Microscopy (GFE), RWTH Aachen University, 52074 Aachen, Germany**

*** Correspondence: a.jalil@fz-juelich.de; Tel.: +49-176-70339392**

Section 1 – Fabrication of patterned Substrates

The fabrication of the patterned substrates is conducted in the following steps:

- **Preparation of the blocking surfaces:** A 4-inch Si (111) wafer is cleaned with Piranha. The native SiO_2 is removed via wet etching using 1 % HF solution. The wafer is then loaded into the Tempress oxidation furnace and 5-7 nm thick SiO_2 layer is prepared via dry oxidation process at 900 °C. The oxidized wafer is then loaded into the Oxford PECVD chamber and 25-30 nm of Si_3N_4, using the mixed frequency procedure, is deposited. The mixed frequency deposition assists in controlling the strain of the deposited film. The PECVD deposited Si_3N_4 is unstable in HF based solutions due to the presence of H^+ ions in the film. In order to increase the stability of Si_3N_4, the deposited wafer is processed through rapid thermal annealing (RTA) to harden Si_3N_4 layer. The annealing profile is depicted in Figure S1. After the annealing process, the etching rate of PECVD Si_3N_4 dropped from 25 nm/min to approx. 2 nm/min in the buffered HF.
- **Lithography for pattern transfer:** The processed wafer, discussed above, is cleaned with solvents and spin coated for the first lithography step to prepare the global alignment markers on the wafer. Via e-beam lithography (EBL) and

reactive ion etching (RIE) with CHF_3/SF_6 gases, 700 nm deep structures are etched (negative markers) into the Si wafer that provide a platform for precise alignment, with a tolerance of ±5 nm, for future lithography cycles. In the next step, the pattern transfer for selective growth is performed. Once again using EBL, patterns with the desired shape and dimensions are projected onto the substrates that are finalized via dry etching using CHF_3 and O_2 gases with precisely etching Si_3N_4 layer leaving behind trenches with Si (111) surfaces buried under a thin layer of SiO_2. The exact parameters for each lithography and etching steps can be found here [1]. Before epitaxy, the buried Si (111) surfaces are released by etching SiO_2 using HF solution. SEM images of a few finalized patterned substrates after removing SiO_2 are shown in Figure S2.

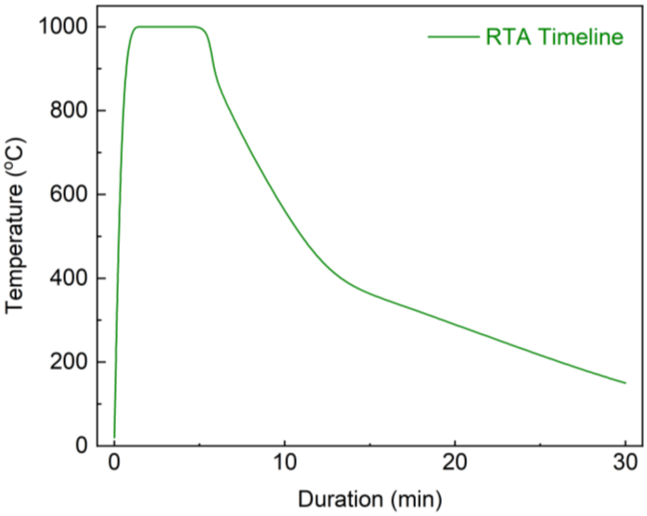

Figure S1. RTA timeline representing all three process steps including ramp-up, bake-out and cool-down of Si_3N_4 annealing process.

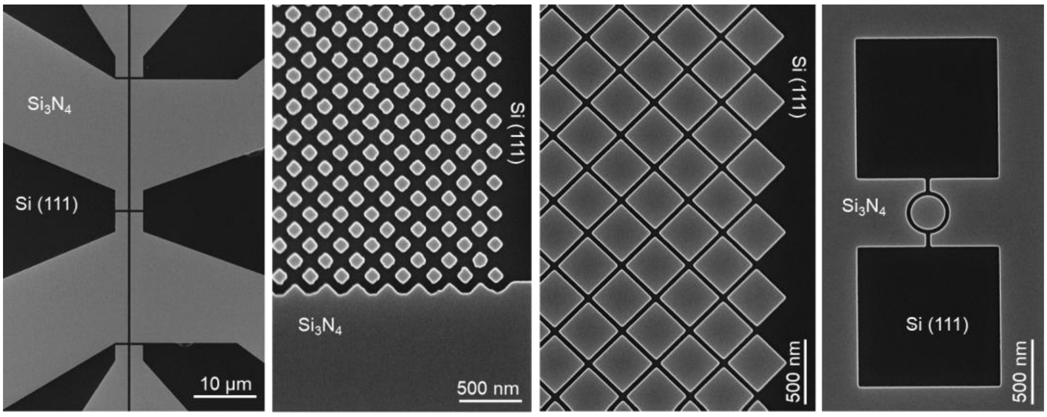

Figure S2. SEM images of several patterned substrates where the dark contrast represents the epitaxial surfaces inside the etched trenches after removing Si_3N_4 via RIE and SiO_2 via HF solution while the bright contrast represents the blocking surfaces.

Section 2 – Growth optimization

<u>*Surface treatment:*</u> The HF etched patterned substrate is loaded into the MBE chamber and heated at 700 °C for 20 minutes to degas the passivated hydrogen atoms from the surface. The substrate is then cooled down to the growth temperature where the Si dangling bonds are exposed to the Te beam flux that results in the formation of 1x1 Te monolayer. This Te termination layer provides a base for the *van der Waals* epitaxy of 3D TIs.

<u>*Growth temperature:*</u> At first, the search for the optimum growth temperature (T_{sub}) is conducted. The study is started with binary TIs where the epitaxy is conducted over a vast

range of T_{sub} from 250 ˚C to 330 ˚C with the different applied growth rates (R_{TF}). An overview of Bi_2Te_3 growth is presented in Figure S3a where two different Bi effusion cell temperatures (T_{Bi}), indicated by blue (T_{Bi} = 475 ˚C) and red (T_{Bi} = 490 ˚C) colors, result in the respective growth rates of 12.3 nm/h and 17.1 nm/h. It is also important to mention here that it is only the Bi beam flux (T_{Bi}) that controls the growth rate of the epilayer, as depicted in Figure S3a, providing enough Te flux is available to form the compound. Te beam flux does not affect the growth rate and is always provided in excess. Later, detailed XRD analysis is performed on the grown epilayers where the full width at half maximum (FWHM) of the rocking curve, acquired at the (0 0 15) peak is utilized as a qualitative figure of merit. A trend of Bi_2Te_3 RC analysis, corresponding to Figure S3a, is depicted in Figure S3b.

Temperature zones: Based on RC analysis, the entire temperature range is divided into separate sections. From T_{sub} = 250 ˚C to 275 ˚C, epilayers with the rocking curve values higher than 500 arcsecond are acquired. This temperature range is named the high defect density zone and represented with orange colored region in Figure S3b. Between 275 ˚C and 295 ˚C a continuous improvement in RC values is observed and thus, this range is named the transition zone. In the narrow temperature range, between 295 ˚C and 305 ˚C, epilayers with the lowest rocking curves FWHM values, below 150 arcsecond, are achieved. This range is called the optimum zone, represented with the green color, and is the most important for future growth steps. As soon as T_{sub} reached higher than 305 ˚C, the adsorption to desorption ratio dropped significantly resulting in the non-coalesced epilayers. This temperature range is named the deformation zone and is indicated with a red colored region in Figure S3b. In similar fashion, growth studies for Sb_2Te_3 and BST alloys are conducted and the corresponding optimum zones are identified.

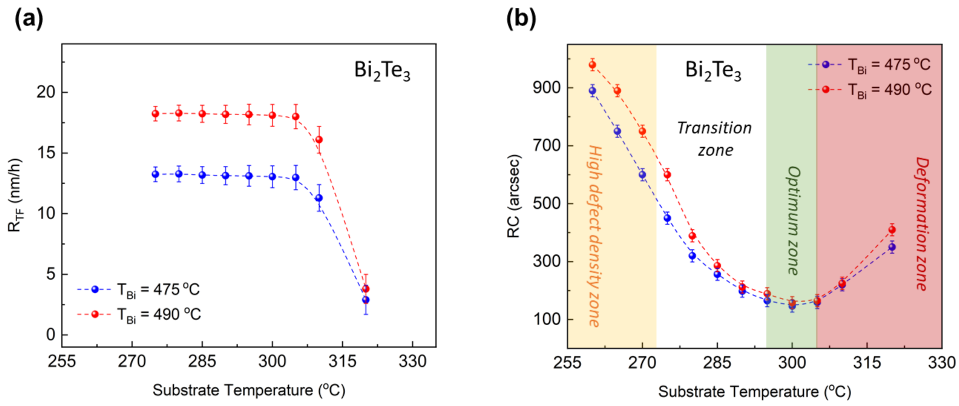

Figure S3. Growth rate (R_{TF}) vs. T_{sub} behavior of (a) Bi_2Te_3 and (b) the corresponding FWHM values of the rocking curve acquired at the (0 0 15) peak of the crystal. The Yellow, white, green and red colors represent the high defect density, transition, optimum and deformation zones respectively.

Growth rate: In the next step, T_{sub} is fixed to the optimum vale of 300 ˚C and the growth rate (R_{TF}) is varied to investigate the effects on the crystal quality of the epilayers. It is observed that the crystal quality improves significantly when R_{TF} is reduced to 5 nm/h or blow. The best FWHM value of the rocking curve i.e. 145 arcsecond, represented in Figure S3b at T_{sub} = 300 ˚C, is further improved to 68 arcsecond, for Bi_2Te_3, when R_{TF} is reduced to 5 nm/h while T_{sub} remained unchanged. The measured XRD patterns of Bi_2Te_3, Sb_2Te_3 and $Bi_{0.5}Sb_{1.5}Te_3$ epilayers, prepared at 5 nm/h, are depicted in Figure S4 (a-c) while an example of the acquired RC at the (0 0 15) peak is depicted in Figure S4d.

Twin suppression: One of the other advantage of lowering R_{TF} to 5 nm/h or below is the suppression of rotational twin domains in the epilayer. All 3D TIs, included in this study, exhibit the trigonal crystal structures. While growing on Si (111) substrate with a hexagonal surface, starting nucleation has a 60° rotational freedom to align itself with the

substrates. This phenomenon leads to the formation of rotational twin domains. Twin domains are observed to be collinear with Si (220) and/or Si (311). These twin defects eventually cause the formation of other extended defects such as grain boundaries and translation sheer faults. It has been observed that epilayers prepared with R_{TF} (>> 5 nm/h) exhibit a high density of such defects that can be suppressed by lowering R_{TF}. An example to twin suppression in Bi_2Te_3 epilayers is shown in Figure S5 where the XRD φ-scans of the (1 0 5) peak represent the relative presence of twin defects in epilayers that are prepared with different R_{TF}. One of the reason of acquiring lower values of RC while $R_{TF} \leq 5$ nm/h, observed in Figure S4d, is the presence of low density of extended defects in the epilayer.

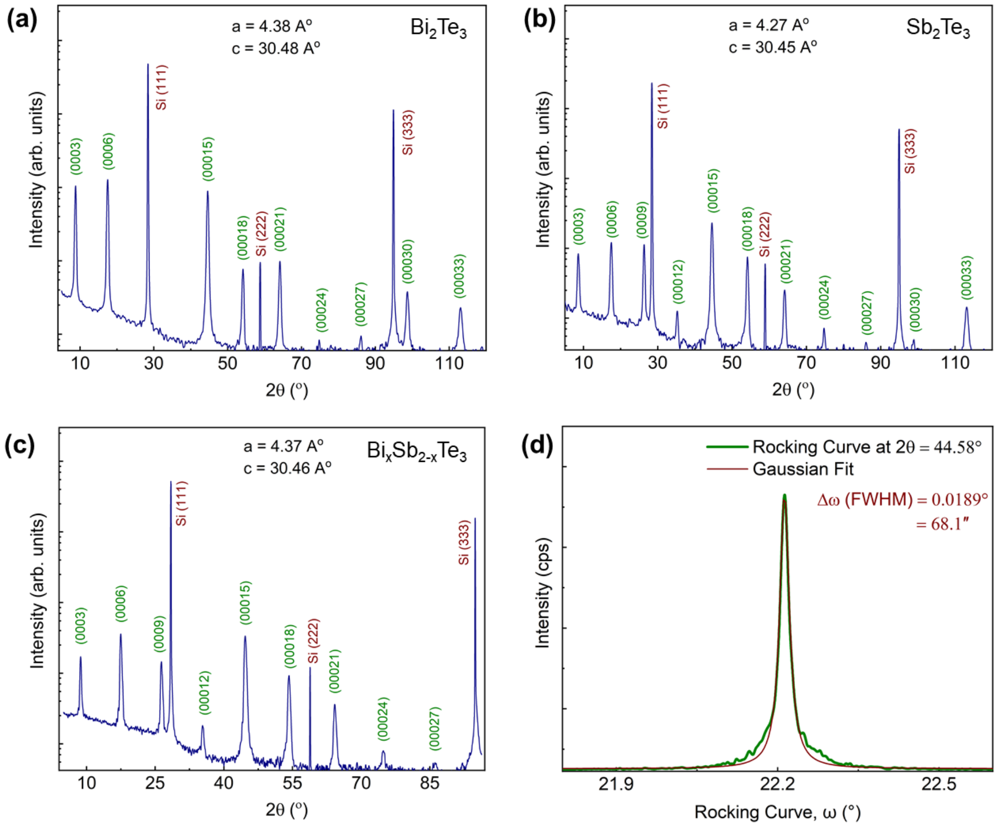

Figure S4. XRD *2θ-ω* scans of approx. 30nm thick epilayers of (a) Bi_2Te_3, (b) Sb_2Te_3 and (c) $Bi_{0.5}Sb_{1.5}Te_3$ alloy. The relative intensity of the (0 0 0 9) diffraction peak in BST alloy (c) indicates the presence of higher Sb contents. (d) Displays the rocking curve ($\Delta\omega$) scan of Bi_2Te_3, acquired at the (0 0 0 15) peak, along with Gaussian fitting.

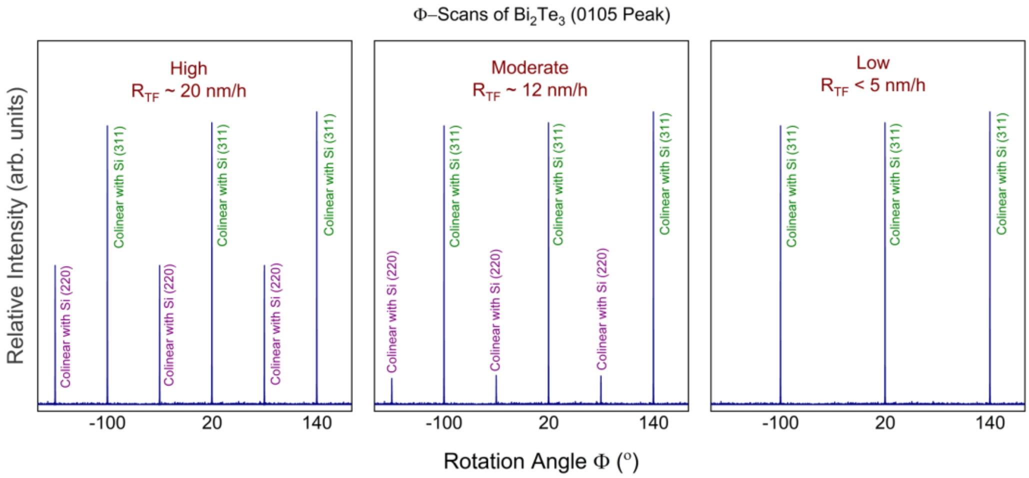

Figure S5. Analysis of rotational twin domains via XRD φ-scan for Bi_2Te_3 epilayers acquired at the (1 0 5) peak along with their relative intensity collinear with Si (311) and (220) orientations. (a) Epilayers prepared at higher R_{TF} exhibited high density of twin defects. (b) Suppressed twin domains along Si (220) with the relative abundance of 1:9 is observed in epilayers that were prepared at moderate R_{TF}. (c) Twin free epilayers collinear only with Si (311) are obtained when prepared at low R_{TF}.

<u>*Effect of Te beam flux:*</u> Before proceeding to selective growth, the investigation of Te flux change and its impact on the epilayer quality is crucial. As mentioned earlier, Te beam flux (T_{Te}) does not influence R_{TF} and therefore, it is kept constant throughout this study at T_{Te} = 330 °C. One of the immense challenge in the growth optimization of 3D TIs is addressing the point defects. Among them, the most critical is the Te vacancies (V_{Te}). To lower the density of V_{Te}, Te beam flux is kept high enough to make sure that the flux ratio between the rate controlling elements (Bi, Sb) and Te always remains 1:10 or higher. By lowering the flux ratio, the probability of V_{Te} increases while the increment in the ratio results in increasing the surface roughness of the epilayer. In short, as far as the flux ratio is kept high enough to affirm the stoichiometric growth of 3D TIs, Te flux does not impact R_{TF}. Figure S6 depicts the observable effect in the epilayer by lowering the Te flux as far as the flux ratio does not cross the limit; however, after crossing the limit the epilayer results in the formation of stoichiometric alloy such as Bi_xTe_y and Sb_xTe_y, a study of which is outside the scope of this work.

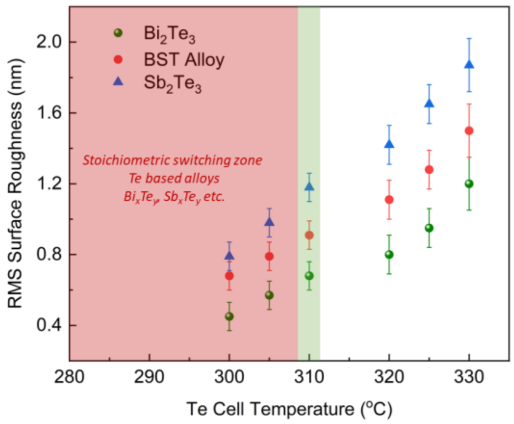

Figure S6. The trend of average surface roughness with changing Te flux. The red zone indicates the critical T_{Te} where Bi/Te or Sb/Te flux ratios cross the limit and result in the altered

stoichiometry. The green zone indicates the lowest possible T_{Te} that provides the best (lowest) surface roughness while keeping the stoichiometry intact.

Section 3 – Selective temperature zone

As introduced in the main text, selectivity is a temperature sensitive technique that depends upon the adsorption to desorption ratio (ADR) of adatoms on the surface. Bi and Sb adatoms exhibit different ADR trend on Si (111) surface with changing T_{sub}.

- The Bi adatoms exhibit strong adhesion (adsorption coefficient) and that is why, ADR of Bi remains almost constant throughout the entire T_{sub} range i.e. R_{TF} remains almost constant, until the deformation zone is reached as depicted in Figure S7a.
- Sb adatoms exhibit weak adhesion to the substrate and a strong dependency on T_{sub}. It results in continually decreasing R_{TF} with increasing T_{sub} confirming an inversely proportional relation as depicted in Figure S7b.

The behavior of both elements is observed to be similar on the blocking surface of Si_3N_4 as observed on Si (111).

- The Bi adatoms exhibit strong adhesion, even on the blocking surface and therefore, a very narrow selective zone is observed as depicted in Figure S7a. As desired, the selective zone resides within the optimum zone, similar to BST alloys, depicted in Figure 2.
- The Sb adatoms exhibit poor adhesion and thus low ADR on the blocking surface. This phenomenon ensures a relatively large selective zone as depicted in Figure S7b. Though, the selectivity for Sb_2Te_3 crystals can easily be achieved; the entire selective zone is not usable to acquire high crystal quality structures as a decently large part of it resides outside the optimum temperature zone. The high quality selective growth can only be achieved in a narrow window where the optimum and the selective zones overlap.

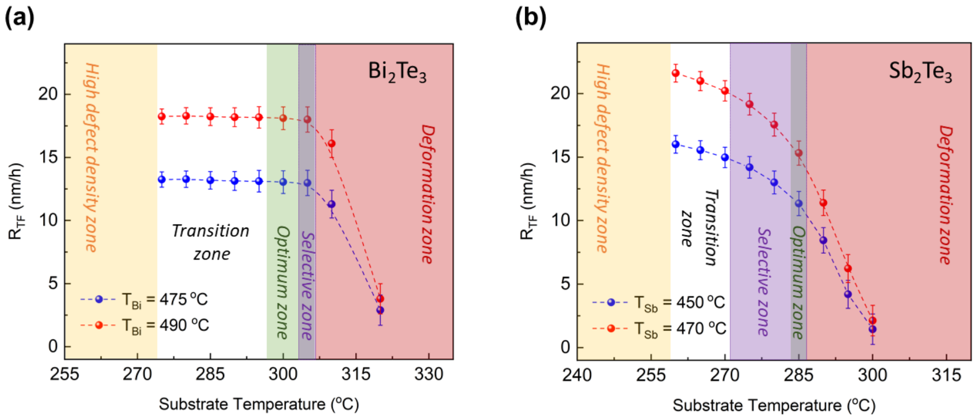

Figure S7. Growth rate (R_{TF}) vs. T_{sub} behavior of (a) Bi_2Te_3, (b) Sb_2Te_3 for the identification of optimum and selective temperature zones.

Section 4 – Thickness of selectively grown nanostructures

The thickness of the TI film in the selectively grown nanostructures is measured using the following three methods:

- <u>*Cross-sectional SEM:*</u> The selectively grown nanostructures are etched via focus ion beam (FIB) and at the cross-section of the nanoribbon, SEM images are acquired

that have provided a rough estimate of the layer thickness (with ± 1 nm accuracy). Details can be found in *Rosenbach et al.* [2].

- <u>*TEM and STEM*:</u> A second approach adopted to measure the thickness of TI layer is the transmission electron microscopy (TEM) and scanning TEM (STEM). This is relatively an expensive and time consuming approach that cannot be utilized on the regular basis; however in this study, it is adopted to investigate the atomic-scale structural characterization of nanostructures after the growth optimization of each material system. This approach provides the high resolution measurements precise to a sub-nm scale. An example of this approach is depicted in Figure S8 where a couple of HAADF STEM images indicate the QL stacking of Bi_2Te_3 and Sb_2Te_3 epilayers respectively along with the precise measurement of the layer thickness.
- <u>*AFM:*</u> One of the easily adoptable and relatively accurate technique than SEM is the atomic force microscopy (AFM). In order to evaluate the layer thickness, AFM measurements are conducted on the pre-patterned substrates before and after the MBE growth. The difference in the trench depth provides the exact thickness of the TI epilayer in the particular nanostructure. A couple of examples containing AFM topographical images after SAE are depicted in Figure S9, while Figures S10 – S12 depict several examples of the line scans measurements of the selected regions in the topographical images before and after SAE. In order to acquire accurate measurements, the geometry of the substrate is taken into account and special tips (AR10-NCHR and AR5T-NCHR) with high aspect ratio with approx. (1:15) are used.

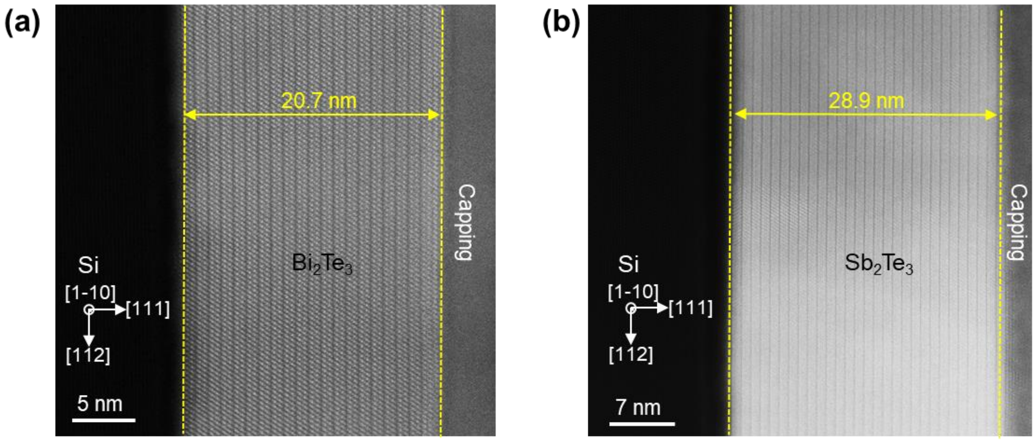

Figure S8. STEM-HAADF images of (a) Bi_2Te_3 and (b) Sb_2Te_3, acquired at the cross-section of 500 nm wide selectively grown nanoribbons along Si [1-10] orientation.

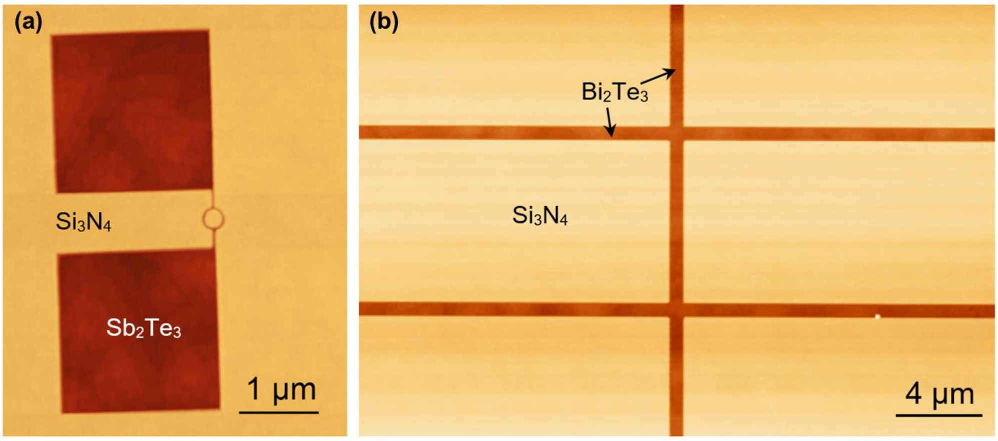

Figure S9. AFM topographical images of selectively grown crystals of (a) Sb_2Te_3 and (b) Bi_2Te_3 on the Si (111) pre-patterned substrates.

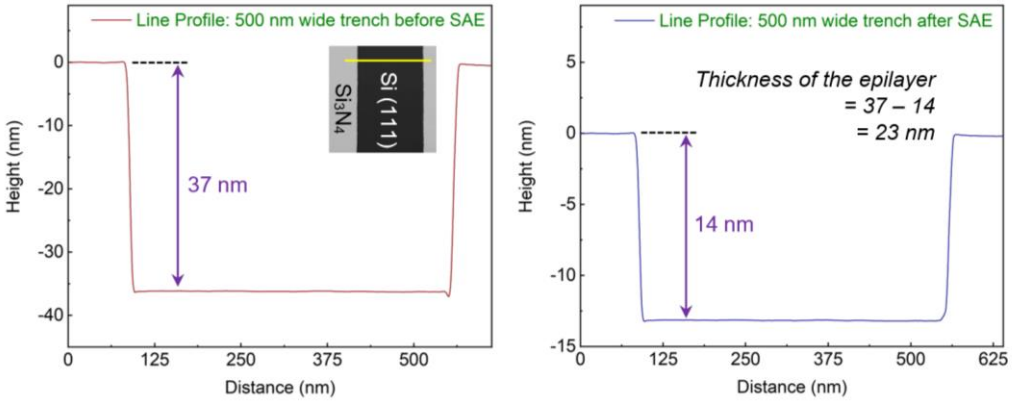

Figure S10. The topographic line scans measured via AFM tapping mode in a 500 nm wide structure before and after the SAE of Bi_2Te_3 that provide the quite accurate measurement of the layer thickness.

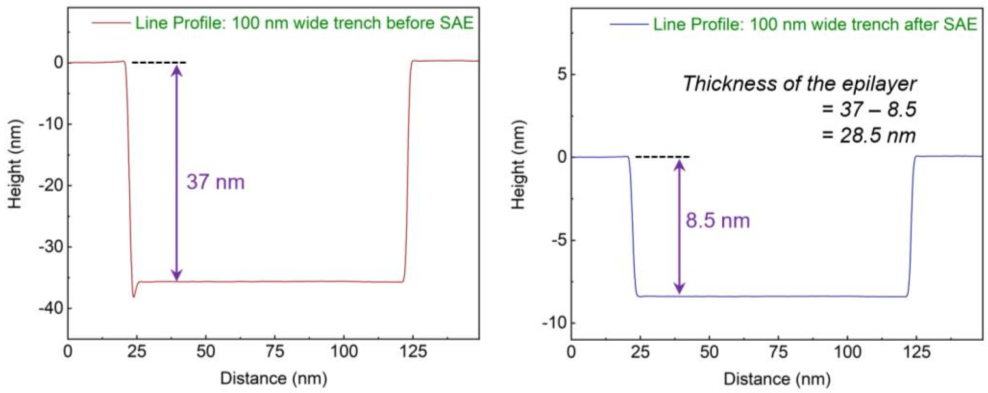

Figure S11. The topographic line scans measured via AFM tapping mode in a 100 nm wide structure before and after the SAE of Sb_2Te_3.

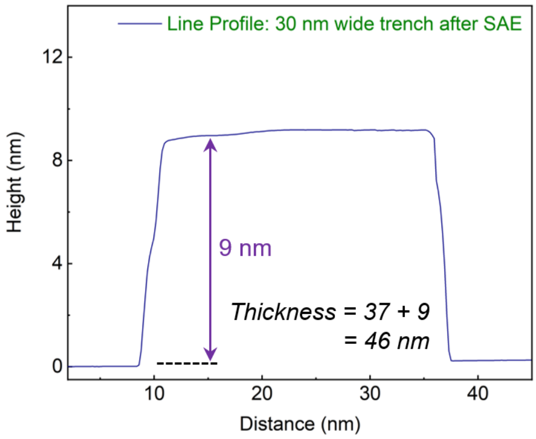

Figure S12. The topographic line scan measured via AFM tapping mode in a 30 nm wide structure after the SAE of the BST alloy that indicates the excessive overgrowth in the nanostructure.

Section 5 – SAE growth model for circular objects and rings

The growth model presented in the main text can be adopted for any desired geometry. In the main text, the model is represented specifically for the rectangular and square objects. Here, two examples for the non-conventional geometries are presented.

Circular Objects: Consider a circular trench as represented in Figure S13 (left). During SAE, the impact of lateral diffusion of adatoms (L_D) will impact the geometry as represented in Figure S13 (right).

The effective growth rates (R_{eff}) can be evaluated using the following equations.

$$A = \pi R^2$$

$$A_{eff} = \pi(R + L_D)^2$$

$$\frac{R_{eff}}{R_{TF}} = \frac{A_{eff}}{A}$$

$$R_{eff} = R_{TF}\left[1 + \frac{2L_D}{R} + \frac{\pi {L_D}^2}{A}\right] \quad \text{(S1.1)}$$

The value $\frac{\pi {L_D}^2}{A}$ is too small to have any impact until R = L_D, and thus can be ignored. Hence, R_{eff} can be represented as:

$$R_{eff} = R_{TF}\left[1 + \frac{2L_D}{R}\right] \quad \text{(S1.2)}$$

- If R >> 1 µm, R_{eff} = R_{TF}
- If R < 1 µm, R_{eff} can be evaluated using equation S1.2
- If R<< 1 µm, so much that R → 2L_D, R_{eff} can be evaluated using equation S1.1

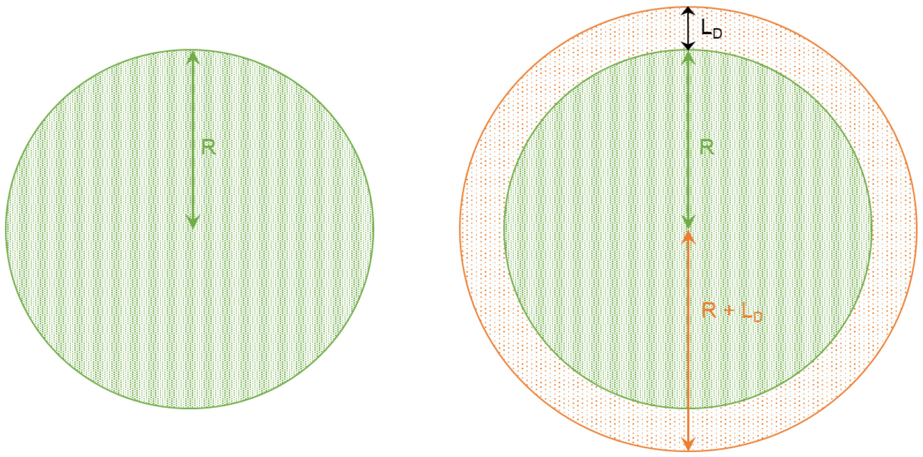

Figure S13. Schematics of a circular trench and the representation of area before and during the selective growth (A_{eff}) due to lateral diffusion length of adatoms (L_D). The radius R of the object increased to (R+L_D) while evaluating A_{eff} during SAE.

<u>*Ring Structures:*</u> Consider a circular ring trench as represented in Figure S14 (left). During SAE, the impact of lateral diffusion of adatoms (LD) will impact the geometry as represented in Figure S14 (right).

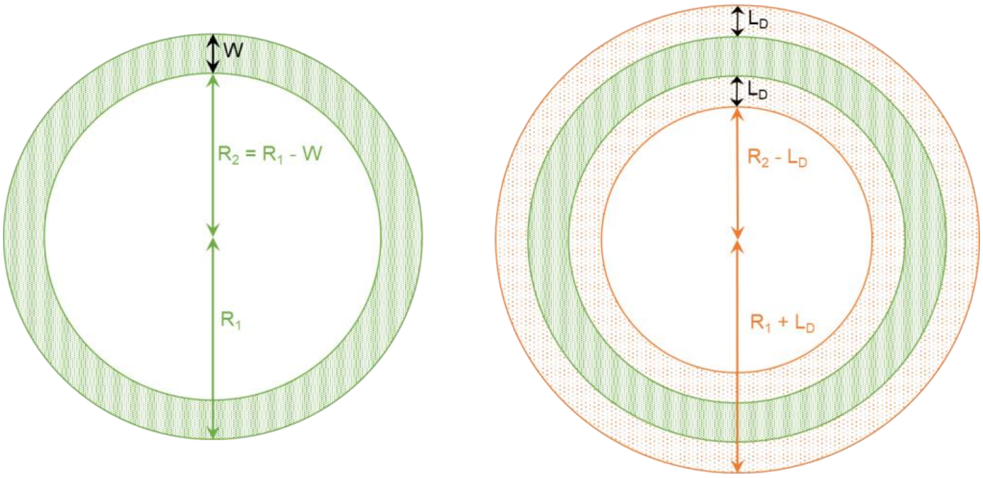

Figure S14. Schematics of a circular ring trench and the representation of area before and during the selective growth (A_{eff}) due to lateral diffusion length of adatoms. The radii of external R_1 and internal rings R_2 of the ring structure will increase to R_1+L_D and R_2-L_D respectively while evaluating A_{eff} during SAE.

The effective growth rates (R_{eff}) can be evaluated using the following equations.

$$A = \pi {R_1}^2 - \pi {R_2}^2 = \pi {R_1}^2 - \pi (R_1 - W)^2$$

$$A = 2\pi R_1 W - \pi W^2$$

$$A_{eff} = \pi (R_1 + L_D)^2 - \pi (R_2 - L_D)^2$$

$$A_{eff} = 2\pi R_1 W - \pi W^2 + 4\pi R_1 L_D - 2\pi W L_D$$

$$\frac{R_{eff}}{R_{TF}} = \frac{A_{eff}}{A}$$

$$R_{eff} = R_{TF}\left[\frac{2\pi R_1 W - \pi W^2 + 4\pi R_1 L_D - 2\pi W L_D}{2\pi R_1 W - \pi W^2}\right]$$

$$R_{eff} = R_{TF}\left[1 + \frac{2L_D}{W}\right] \quad \text{(S1.3)}$$

$$R_{eff} = R_{TF}\left[1 + \frac{2L_D}{(R_1 - R_2)}\right] \quad \text{(S1.4)}$$

- If the width of the ring (W) is >> 1 µm, $R_{eff} = R_{TF}$
- If the width of the ring (W) is < 1 µm, R_{eff} can be estimated via the evaluated relation above.

- In case of ring structures just W is not the only factor responsible for controlling R_{eff}, the individual values of R_1 and R_2 also govern the change.
  - If R_2 will be too small (→ 0), the impact will be slightly smaller and the trend will start to exhibit more close relation to the circular pattern, discussed in equation (S1.2).
  - If R_2 will be large >> 1 μm, the equation (S1.3) will provide the precise evaluation of R_{eff}.

Similarly, any pattern can be adopted to the SAE growth model and the impact of geometrical patterns on R_{eff} can be easily evaluated.

Section 6 – Atomic scale structural characterization

The atomic scale structural characterizations are performed via transmission electron microscopy (TEM) and scanning TEM (STEM). The tools utilized are introduced below:

- <u>TEM:</u> Tecnai G2 F20 field emission TEM is optimized for imaging at medium resolution. For an acceleration voltage of 200 kV a point to point resolution of about 1.4 Å can be reached for optimized parameters. Further technical details can be found here [3].
- <u>STEM:</u> FEI Titan G2 80-200 field emission gun STEM (CHEMI-STEM) is supplied with high brightness Schottky field emission gun. Due to its aberration corrector (monochromator and CS probe corrector) for the condenser lens it is possible to achieve a probe size of 0.8 Å at an accelerating voltage of 200 kV which determines the resolution. For acquiring images an on-axis bright field (BF) and dark field STEM detector is used. Further technical details can be found here [4].

References

1. Jalil, A.R. *Engineering topological superlattices and their epitaxial integration in selectively grown hybrid nanostructures via mbe*; Halbleiter-Nanoelektronik: RWTH Aachen University, 2022; p 309.
2. Rosenbach, D.; Oellers, N.; Jalil, A.R.; Mikulics, M.; Kölzer, J.; Zimmermann, E.; Mussler, G.; Bunte, S.; Grützmacher, D.; Lüth, H., *et al.* Quantum transport in topological surface states of selectively grown bi2te3 nanoribbons. *Advanced Electronic Materials* **2020**, *n/a*, 2000205.
3. Luysberg, M.; Heggen, M.; Tillmann, K. Fei tecnai g2 f20. *Journal of large-scale research facilities JLSRF* **2016**, *2*, A77-A77.
4. Kovács, A.; Schierholz, R.; Tillmann, K. Fei titan g2 80-200 crewley. *Journal of large-scale research facilities JLSRF* **2016**, *2*, A43-A43.
